# Risk prediction models for pregnancy outcomes in recurrent pregnancy loss: a narrative systematic review

**DOI:** 10.3389/fendo.2025.1582156

**Published:** 2025-05-29

**Authors:** Qiliang Jian, Fangxiang Mu, Kexin Wang, Fang Wang

**Affiliations:** Department of Reproductive Medicine, Lanzhou University Second Hospital, Lanzhou, China

**Keywords:** recurrent pregnancy loss, prediction model, pregnancy outcomes, systematic review, general practice

## Abstract

**Objective:**

Recurrent pregnancy loss (RPL) is a significant clinical challenge, with many cases remaining unexplained, and existing risk prediction models often lacking objective evaluation. This study aims to systematically review and evaluate the published risk prediction models for pregnancy outcomes in RPL.

**Methods:**

Literature search was conducted in August 2024 using PubMed, Embase, Web of Science, CNKI, and CMAJ databases to identify studies that reported the development and/or validation of clinical prediction models for RPL pregnancy outcomes. Pregnancy outcomes included pregnancy loss, ongoing pregnancy, and live birth. Data were extracted using the CHARMS checklist. Risk of bias and applicability were evaluated with PROBAST.

**Results:**

A total of 1,112 records were identified, with 15 studies ultimately included, encompassing 22 risk prediction models for evaluating RPL patients’ pregnancy outcomes. The majority were retrospective cohort studies (13/15), and logistic regression was the predominant modeling method (14/15). Sample sizes ranged from 85 to 789, with the number of predictors per model varying from 2 to 18 (median=5). In total, 65 distinct predictors were identified, including five categories: patient-related, imaging-related, thrombophilia-related, metabolic/endocrinologic, and immunological factors, most frequently maternal age (n=10) and number of previous pregnancy losses (n=9). Among the 20 models that reported discriminative performance by the area under the receiver operating characteristics (ROC) curve (AUC), 13 achieved AUC above 0.80 (range: 0.809–0.97). Notably, 7 studies did not perform any form of validation, and only 3 studies conducted external validation. Despite the models reported a good predictive performance, they were all appraised to have high risk of bias in applicability due to methodological deficiencies.

**Conclusion:**

The findings suggest that current risk prediction models for RPL pregnancy outcomes have a high risk of bias in clinical applications, primarily due to methodological flaws in development and validation processes. Future research should focus on data quality, sample diversity, and model transparency to ensure broad applicability across different populations, providing more reliable and effective tools for clinical practice.

**Systematic review eegistration:**

https://www.crd.york.ac.uk/PROSPERO/view/CRD42024570481, identifier CRD42024570481.

## 1 Introduction

Recurrent pregnancy loss (RPL) is commonly referred to two or more spontaneous abortions (1–4). Known factors contributing to RPL include maternal age, previous pregnancy loss, parental chromosomal abnormalities, uterine anatomical abnormalities, endocrine disorders, and immune disorders (5). Despite thorough surveys, the cause of RPL remains unexplained in about 50% of cases (6). Research shows that in nulligravidae, the risk of pregnancy loss escalates with each additional loss, rising from around 11% after three losses to approximately 40% (7). Therefore, developing a tool to predict pregnancy outcomes for RPL women is crucial for better risk assessment and personalized treatment. Clinical risk prediction models utilize medical data and statistical methods to estimate a patient’s future risk of having a certain disease or experiencing an event (8). For RPL, current research primarily focuses on diagnosing and prognosticating outcomes to enhance clinical management (9–11). Despite progress in existing research, many models still lack objective and unbiased validation, particularly external cohort validation, which limits their widespread application and practical value in clinical decision-making. Therefore, this study aims to systematically review the published RPL risk prediction models, aiming to identify the strengths and limitations of existing models and provide recommendations for developing more clinically applicable risk prediction tools in the future.

## 2 Materials and methods

The review was reported in accordance with the Preferred Reporting Items for Systematic Reviews and Meta-Analyses (PRISMA) guidelines (12). The protocol of this systematic review was registered at the International Prospective Register of Systematic Reviews (PROSPERO) (CRD42024570481).

### 2.1 Data sources and search strategy

A comprehensive search was conducted in both English and Chinese databases, including PubMed, Web of Science, Embase, China National Knowledge Infrastructure (CNKI), and China Medical Association Journals (CMAJ), covering studies published up to August 2, 2024. The articles were restricted to Chinese- or English-language literature, as these languages are spoken by large population, and focusing on the most widely used databases in both language communities ensures a comprehensive understanding of the relevant research. Search terms were a combination of controlled vocabulary (Medical Subject Heading [MeSH] terms) and free-text terms, including “abortion, habitual[MeSH terms]”, “recurrent miscarriage”, “recurrent pregnancy loss”, “nomograms[MeSH terms]”, “machine learning[MeSH terms]”, “risk assessment[MeSH terms]”, “risk prediction”, “risk model”, “predictive model”, and “scoring system”. The full search strategies for all databases are provided in **Supplementary Table 1**. Additionally, we screened the reference lists of the included studies and relevant reviews to identify any additional eligible studies that may have been missed during the database search.

### 2.2 Inclusion and exclusion criteria

We included literature that met the following criteria: (1) patients diagnosed with RPL; (2) studies focused on the development and/or validation of risk prediction models for RPL; (3) the prediction model contained at least two predictors; and (4) the pregnancy outcomes limited to pregnancy loss, ongoing pregnancy, or live birth. We excluded the following types of literature: (1) studies that analyzed risk factors without constructing a risk prediction model; and (2) gray literature such as preprints, conference abstracts, reviews, systematic reviews, meta-analyses, editorials, or letters to the editor.

### 2.3 Study selection and data extraction

Two independent researchers, trained prior to the article selection to ensure understanding and consistent application of screening criteria, conducted a step-by-step method to screen each article from the systematic search after removing duplicates. First, the titles and abstracts were used for initial screening. Second, full texts were reviewed to further assess the records that might meet the eligibility criteria. Records that did not match the pre-established criteria were excluded. Any disagreements at each step should be reached through discussion or consultation with a third researcher to reach consensus. Inter-rater agreement will be calculated using Cohen’s kappa (κ) at the title/abstract screening and full-text review stages. After finalizing the included studies, data were extracted using a standardized form based on the checklist for Critical Appraisal And Data Extraction For Systematic Reviews Of Prediction Modeling Studies (CHARMS) by two independent investigators (13). The extracted data included first author, year of publication, study type, study population, study period, sample size, number of events, outcome prediction, candidate and final predictors, missing data, model construction and validation methods, model performance (discrimination and calibration), and model’s final presentation format.

### 2.4 Model risk of bias and applicability assessment

The risk of bias (ROB) and applicability of the included studies were assessed by two other independent investigators trained in the prediction Model Risk Of Bias Assessment Tool (PROBAST) (14, 15), with a third researcher resolving any disagreements. PROBAST assesses ROB across four domains of “Participant Selection,” “Predictors,” “Outcomes,” and “Analysis” using 20 signaling questions. Each question is answered as “yes,” “probably yes,” “no,” “probably no,” or “no information,” and each domain is rated as “high,” “low,” or “unclear” ROB (15). The overall ROB for each study was classified as low, high, or unclear based on these ratings. For instance, a study was rated as having low overall ROB if all domains were rated “low”, while a high ROB was assigned if any domain was rated “high”. If one or more domains were “unclear” but all other domains were rated as “low”, the study was considered to have an unclear ROB.

## 3 Results

**Figure 1:**
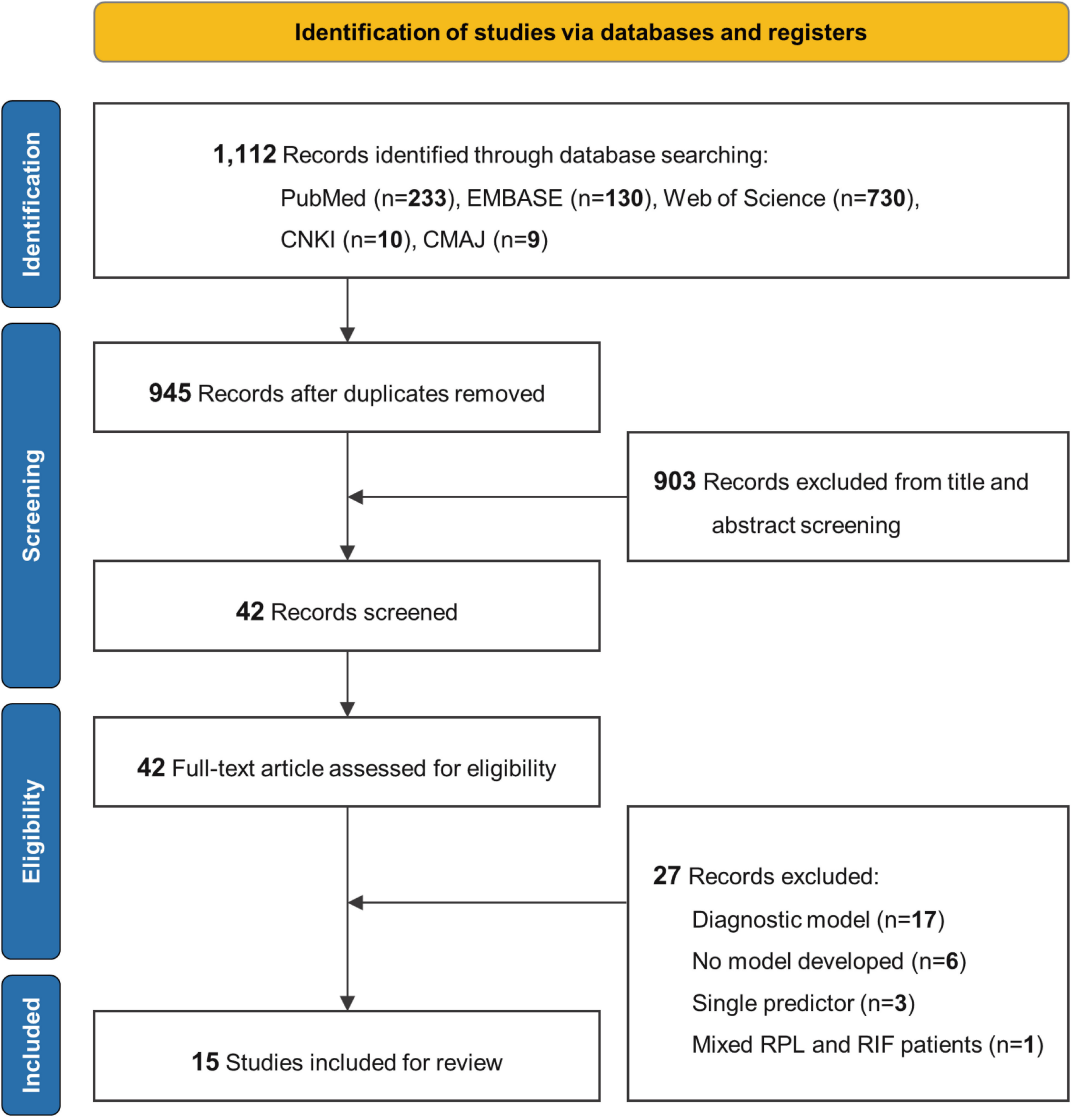
Literature screening process. EMBASE, Excerpta Medica Database; CNKI, China National Knowledge Infrastructure; CMAJ, China Medical Association Journals; RPL, recurrent pregnancy loss; RIF, recurrent implantation failure.

A total of 1,112 papers were identified, 167 of which were duplicates and removed. After screening the 945 records’ titles and abstracts, 42 articles were selected for full-text review. Of these, 17 studies were excluded due to their focus on diagnostic models, 6 studies were excluded because no predictive model was developed, 3 studies only used a single predictor, and 1 study included both RPL and recurrent implantation failure (RIF) patients without providing separate data for RPL cases. Consequently, 15 studies were included in this study (16–30). There was a good inter-rater agreement between the two reviewers for the title/abstract screening (κ = 0.878) and full-text review stage (κ = 0.847). **Figure 1** illustrates the literature screening flowchart.

### 3.1 Basic characteristic

**Table 1** summarizes the characteristics of the 15 included studies. Of these, two were in Chinese (29, 30) and 13 in English (16–28), with all studies were published between 2020 and 2024. In terms of study designs, 13 were retrospective cohort studies (16, 18–23, 25–30), and two were prospective cohort studies (17, 24). The enrollment periods varied, with the longest spanning 15 years (2000~2015) (19, 27) and the most frequent being from 2020 to 2022 (20, 21, 29, 30). The 15 included studies originated from three countries: China (n=12) (16–18, 20–26, 29, 30), the Netherlands (n=2) (19, 28), and Israel (n=1) (27), indicating a predominance of research from China. (**Supplementary Figure 1**).

**Table 1:** Overview of basic data of the included studies.

Author	Year of publication	Country	Enrollment period	Study design	Participants
Bashiri et al. (27)	2022	Israel	2000-2015	Retrospective cohort	RPL
Dai et al. (16)	2022	China	2016-2018	Retrospective cohort	RPL
Fossé et al. (28)	2022	Netherlands	2012-2019	Retrospective cohort	URPL
Geng et al. (29)	2023	China	2020-2022	Retrospective cohort	URPL
Li H et al. (22)	2020	China	2010-2017	Retrospective cohort	RPL
Li Y et al. (20)	2024	China	2020-2022	Retrospective cohort	URPL
Liu et al. (25)	2024	China	2019-2021	Retrospective cohort	RPL
Mu et al. (21)	2024	China	2020-2022	Retrospective cohort	RPL
Mu et al. (23)	2024	China	2019-2022	Retrospective cohort	RPL
Ou et al. (17)	2024	China	2017-2021	Prospective cohort	URPL
Wu (30)	2023	China	2020-2022	Retrospective cohort	RPL
Yang et al. (24)	2024	China	2019-2022	Prospective cohort	RPL
Youssef et al. (19)	2022	Netherlands	2004-2019	Retrospective cohort	URPL
Zhang J et al. (18)	2024	China	2021-2022	Retrospective cohort	TAI-positive RPL
Zhang Z et al. (26)	2024	China	2016-2018	Retrospective cohort	RPL

RPL, recurrent pregnancy loss; URPL, unexplained RPL; TAI, thyroid autoimmunity.

### 3.2 Population and outcomes

As shown in **Table 2**, almost all studies defined RPL as two or more pregnancy losses (16–28, 30), except one study required at least three or more losses (29). The discrepancies in the definitions of RPL mainly regarding the gestational age and whether losses needed to be consecutive. Specifically, 10 studies consistently reported a gestational age of 24 weeks (17–21, 23–25, 27, 28). Two studies used different gestational ages of 28 (16) and 20 weeks (26), while three did not report (22, 29, 30). Furthermore, four studies required consecutive losses, with gestational ages of 28 (16), 24 (17, 18), and 20 weeks (26). The remaining studies did not specify consecutive losses, with most reporting a gestational age of 24 weeks. **Supplementary Table 2** shows participant inclusion and exclusion criteria. For the predictive outcomes, two-third studies (10/15) developed models for pregnancy loss risk, including “pregnancy loss” (16, 20–23, 29, 30) and “early pregnancy loss” (18, 24, 25); one-third studies constructed for “ongoing pregnancy” (19, 28) and “live birth” (17, 26, 27). Notably, the definitions of predicted outcomes varied significantly, with details presented in **Table 2**.

**Table 2:** Definitions of RPL and predicted outcomes in the included studies.

Included studies	Definition of RPL			Definition of predicted outcome	
	Number of PPL	Consecutive	GA	Outcome	Definition
Bashiri (2022) (27)	≥2		24	LB	Pregnancy resulting in the livebirth of a baby, over 24 weeks of gestation
Dai (2022) (16)	≥2	✓	28	PL	Spontaneous abortion before 28 weeks of gestation, including stillbirth, embryo damage, biochemical pregnancy, etc.
Fossé (2022) (28)	≥2		24	OP	Fetal survival beyond 24 weeks of gestation in the first pregnancy after intake consultation
Geng (2023) (29)	≥3		NR	PL	–
Li H (2020) (22)	≥2		NR	PL	Absence of a previously positive EHM determined by more than two transvaginal or pelvic ultrasonography scans and incomplete or complete expulsion of the embryo after vaginal bleeding
Li Y (2024) (20)	≥2		24	PL	Spontaneous abortion (the termination of a pregnancy before 24 weeks gestation, with fetal weight <1000 g) and biochemical losses (characterized by elevated levels of hCG in blood or urine, despite the absence of a gestational sac confirmed by gynecological color Doppler ultrasound examination)
Liu (2024) (25)	≥2		24	EPL	Pregnancy loss before 13 weeks of gestation
Mu (2024) (21)	≥2		24	PL	Spontaneous abortion before 24 weeks of gestation
Mu (2024) (23)	≥2		24	PL	Spontaneous loss of pregnancy before 24 weeks of gestation
Ou (2024) (17)	≥2	✓	24	LB	Delivery of at least 1 live baby after 24 weeks’ gestation
Wu (2023) (30)	≥2		NR	PL	Pregnancy was terminated before 28 weeks of gestation or the fetal weight <1000 g
Yang (2024) (24)	≥2		24	EPL	Pregnancy less than 10 weeks of gestational age, including biochemical pregnancy
Youssef (2022) (19)	≥2		24	OP	Heartbeat detected via ultrasound, over 24 weeks of gestation
Zhang J (2024) (18)	≥2	✓	24	EPL	Pregnancy loss before 12 weeks
Zhang Z (2024) (26)	≥2	✓	20	LB	The occurrence of uneventful pregnancies that were spontaneously conceived, resulting in the delivery of a live baby following CsA treatment

PPL, previous pregnancy losses; NR, not reported; GA, gestational age (in weeks); LB, live birth; PL, pregnancy loss; OP, ongoing pregnancy; EPL, early pregnancy loss; EHM, embryonic heart motion; hCG, human chorionic gonadotropin; CsA, cyclosporine A.

### 3.3 Predictors

**Table 3** shows the information on the models’ construction. Fourteen studies reported their method for selecting predictors, with common methods including the Least Absolute Shrinkage And Selection Operator (LASSO) selection (20, 21, 23, 24), backward stepwise logistic regression (21, 22, 26, 28), and univariate analysis (16, 25, 27, 29, 30). The number of predictors in the final model ranged from 2 to 18, with a median of 5.

**Table 3:** Information on the construction of prediction models.

Included studies	Sample size (T/V)	Number of events/EPV	Missing data number/processing	Construction methods	Model presentation	Candidate/final predictors (n)	Method for selection of predictors	Final models
Bashiri (2022) (27)	675/-	484/47.8	-/NR	Multivariable Logistic regression	–	4/4	Univariate analysis	Age, number of pregnancy losses, RPL workup, type of pregnancy loss
Dai (2022) (16)	242/-	34/11.3	-/-	Multivariable Logistic regression	Scoring system	3/3	Univariate analysis	ANA spectrum, PS, aPL
Fossé (2022) (28)	526/-	345/20.1	6~61/multiple imputation	Multivariable Logistic regression	Formula	9/7	Backward elimination	Number of previous pregnancy losses, maternal age, paternal age, maternal BMI, paternal BMI, maternal smoking status, mode of conception
Geng (2023) (29)	136/-	52/5.8	-/complete-case analysis	Multivariable Logistic regression	Nomogram	9/4	Univariate analysis	Number of spontaneous abortions, sFlt-1, D-Dimer, ultrasound multimodal score
Li H (2020) (22)	789/-	164/9.1	-/NR	Multivariable Logistic regression	–	18/5	Backward stepwise logistic regression	Gravidity, abdominal pain, vaginal bleeding, base-10 log-transformed peak serum β-hCG, progesterone
Li Y (2024) (20)	374/128	-/-	65/multiple imputation	Logistic regression	Nomogram, on-line calculator	49/18	LASSO selection	Model 1: age, number of miscarriages, C3, C4, IgA, LDL, TG, HDL/TC, β2GPI-IgM, pT(%), pT/Ts(%), pNK(%), pNK(No.), ANA, anti-ribosomal P, anti-Sm, LA1, ANGLE Model 2: age, C3, C4, LDL, TG, pNK(%), pNK(No.) Model 3: number of miscarriages, C3, C4, LDL, TG, pT(%), pNK(%), pNK(No.), ANA Model 4: age, number of miscarriages, C3, C4, TG, pT(%), pNK(%), pNK(No.)
Liu (2024) (25)	603/-	103/11.4	<121/multiple imputation	Multivariable Logistic regression	–	9/5	Univariate analysis	Models for 5/6/7/8/9^th^ week of gestation: 5^th^ week: age, progesterone 6^th^ week: age, mGSD, CRL 7^th^ week: age, hCG, CRL 8^th^ week: CRL 9^th^ week: mGSD, CRL
Mu (2024) (21)	575/272	171/9.5	-/multiple imputation	Multivariable Logistic regression	Nomogram, on-line calculator	18/7	LASSO selection, backward stepwise regression	BMI, number of previous pregnancy losses, T3, FT4, TSH, LY30, EPL
Mu (2024) (23)	357/92	119/2.6	-/complete-case analysis	Multivariable Logistic regression	Nomogram	45/10	LASSO selection	Maternal age, age of menarche, previous pregnancy loss, IL-10, C4, IgA, aPT-IgG/IgM, RF-IgA, LA1/LA2 ratio
Ou (2024) (17)	176/76	-/-	-/complete-case analysis	Multivariable Logistic regression	Nomogram	16/4	Pairwise correlations<0.7, Akaike information criteria	BMI, pNK, AMH, parity
Wu (2023) (30)	105/-	27/1.0	-/NR	Multivariable Logistic regression	–	27/3	Univariate analysis	Age, BMI, TSH
Yang (2024) (24)	454/151	140/2.2	<40/multiple imputation	Machine learning	Nomogram	63/9	LASSO selection	Age, BMI, history of induced abortion, number of pregnancy losses, ACA, IgM, HCY, PNR, LHR
Youssef (2022) (19)	739/-	474/132.5	-/complete-case analysis	Multivariable Logistic regression	Formula	2/2	Pre-specified model (no selection)	Age, number of pregnancy losses
Zhang J (2024) (18)	85/-	22/0.7	-/complete-case analysis	Multivariable Logistic regression	Formula	33/7	Random forest	Age, IL-6, TT3, IFN-γ, total bilirubin, number of previous miscarriages, C1q
Zhang Z (2024) (26)	154/-	106/2.1	-/complete-case analysis	Multivariable Logistic regression	–	23/5	Stepwise selection	Age, ANA, ACA, aβ2GPI, Ig

A total of 65 distinct predictors were identified, categorized into five categories: (1) Patient-related (e.g., maternal/paternal age, maternal body mass index [BMI], number of pregnancy losses); (2) Imaging-related (e.g., mean of the gestational sac diameter, crown-rump length); (3) Thrombophilia-related (e.g., D-dimer, protein S); (4) Metabolic/endocrinologic (e.g., progesterone, thyroid stimulating hormone [TSH], anti-Müllerian hormone); and (5) Immunological factors (e.g., antinuclear antibody [ANA], anti-cardiolipin antibody [ACA], complement 4 [C4]). A full list of predictors is presented in **Supplementary Figures 2**, **3**.

**Figure 2:**
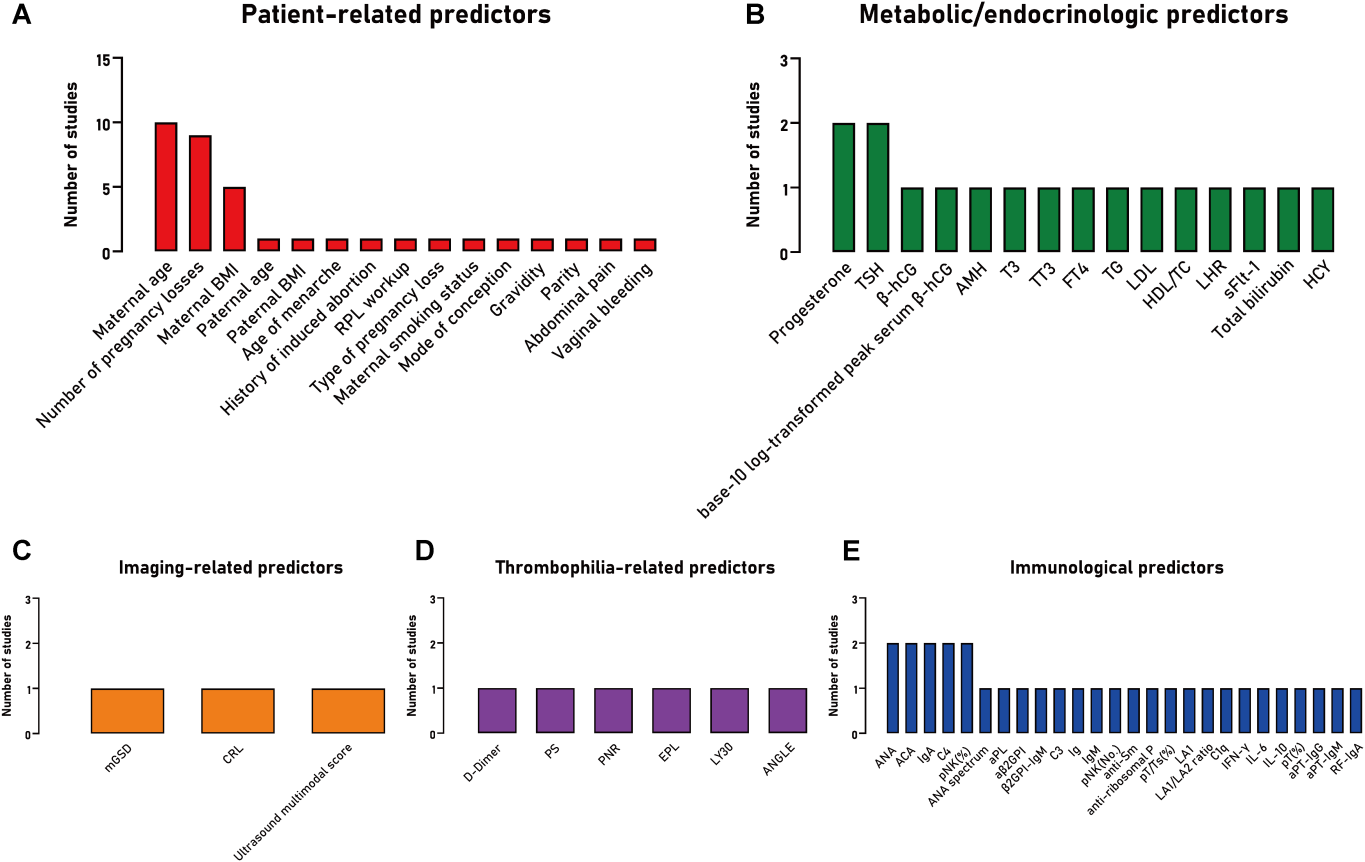
The frequency of predictors included in studies developing a clinical prediction model for the prognosis of patients with recurrent pregnancy loss, stratified by predictor type. **(A)** Patient-related predictors; **(B)** Metabolic/endocrinologic predictors; **(C)** Imaging-related predictors; **(D)** Thrombophilia-related predictors; **(E)** Immunological predictors. BMI, body mass index; RPL, recurrent pregnancy loss; TSH, thyroid stimulating hormone; β-hCG, β human chorionic gonadotropin; AMH, anti-Müllerian hormone; T3, triiodothyronine; TT3, total triiodothyronine; FT4, free thyroxine; TG, triglyceride; LDL, low-density lipoprotein; HDL, high-density lipoprotein; TC, cholesterol; LHR, low-density lipoprotein to high-density lipoprotein ratio; sFlt-1, soluble vascular endothelial growth factor receptor-1; HCY, homocysteine; mGSD, mean of the gestational sac diameter; CRL, crown-rump length; PS, protein S; PNR, platelet to neutrophilic ratio; EPL, an estimated percentage of clot lysis at 30 minutes after the maximum amplitude on TEG tracing; LY30, an actual percentage of clot lysis at 30 minutes after the maximum amplitude on TEG tracing; ANGLE, angle Alpha; ANA, antinuclear antibody; ACA, anti cardiolipin antibody; IgA, immunoglobulin A; C4, complement 4; pNK, peripheral natural killer cells; pNK (%), percentage of pNK; pNK (No.), number of pNK; aPL, anti-phospholipid antibody; β2GPI, β2 glycoprotein I antibody; aβ2GPI, anti-β2 glycoprotein I antibody; C3, complement 3; Ig, immunoglobulin; IgM, immunoglobulin M; IgG, immunoglobulin G; pT, peripheral T cells; Ts, suppressor T cells; pT (%), percentages of peripheral T cells; LA1, lupus anticoagulant screening test; LA2, lupus anticoagulant confirmatory test; C1q, complement component 1q; IFN-γ, interferon-γ; IL-6, interleukin-6; IL-10, interleukin-10; aPT, anti-prothrombin antibody; RF, rheumatoid factor.

The most frequently identified predictors included the model were patient-related: maternal age (n=10), number of pregnancy losses (n=9), maternal BMI (n=5); followed by metabolic/endocrinologic (n=2: progesterone, TSH) and immunological factors (n=2: ANA, ACA, C4, immunoglobulin A [IgA], percentage of peripheral natural killer [pNK] cells) (**Figure 2**).

### 3.4 Missing data

Fourteen studies acknowledged the presence of missing data during the development of their risk prediction models (**Table 3**). In specific, 5 studies handled missing data using multiple imputations (20, 21, 24, 25, 28), while 6 studies performed complete case analysis by excluding individuals with missing data (17–19, 23, 26, 29). However, 3 studies reported missing data without specifying the handling method (22, 27, 30), and one study did not report any information regarding missing data (16). Since statistical software defaults to complete case analysis when the handling of missing data is unspecified (31), we assumed that these studies employed complete-case analysis.

### 3.5 Model construction

Fifteen studies encompassed 22 prediction models, with candidate predictors ranging from 2 to 63 and sample sizes between 85 and 789. The events per variable (EPV) varied from 0.7 to 132.5. Logistic regression was the primary approach (16–23, 25–30), while one study employed machine learning algorithms (24). For model presentation, one model used a risk scoring system, six used nomograms, three presented logistic regression equations, and two were online calculators. It should be noted that in some studies containing multiple models (20, 25), these models shared the same dataset, candidate variable selection process, and construction procedures, differing only through distinct predictor combinations to achieve prediction objectives. Details are in **Table 3**.

### 3.6 Model evaluation

The discriminative ability was assessed with the area under the receiver operating characteristics (ROC) curve (AUC) in 13 studies and C-statistics in 2 studies (**Table 4**). Among the 20 models that provided AUC values, 13 of them exhibited an AUC exceeding 0.80 (ranges from 0.809~0.97), while the remainder indicated moderate discrimination, ranging from 0.62 to 0.796. Additionally, 10 studies assessed how well the predicted risks compared to the observed risks (calibration), with 7 conducting the Hosmer-Lemeshow goodness-of-fit test (17, 18, 20, 21, 23, 24, 29), 9 presenting calibration plots (17–21, 23, 24, 28, 29), and 3 employing the calibration slope (17, 19, 28).

**Table 4:** Information on the performance of prediction models.

Included studies	Internal validation	External validation	Discrimination	Calibration	
				Plot	Statistics
Bashiri (2022) (27)	–	–	C-statistic: 0.62 (0.57-0.66)	–	Calculation according to the percentage of live birth in accordance with the total score grade
Dai (2022) (16)	–	–	AUC: 0.733 (0.637-0.828)	–	–
Fossé (2022) (28)	Bootstrap	–	AUC: 0.6563 (Apparent), 0.6266 (bootstrap)	✓	Adjusted calibration slope=0.77
Geng (2023) (29)	Bootstrap	–	AUC: 0.933 (0.877-0.969)	✓	HL: χ^2^ = 0.322, *P*=0.113
Li H (2020) (22)	–	–	AUC: 0.81 (0.78-0.84)	–	–
Li Y (2024) (20)	Random split data (7:3)	**-**	Model 1: AUC(T)=0.97 (0.95-0.99), AUC(V)=0.86 (0.83-0.90) Model 2: AUC(T)=0.96 (0.93-0.99), AUC(V)=0.88 (0.84-0.91) Model 3: AUC(T)=0.96 (0.93-0.99), AUC(V)=0.88 (0.85-0.91) Model 4: AUC(T)=0.97 (0.94-0.99), AUC(V)=0.88 (0.85-0.92)	✓	Model 1: HL(T): χ^2^ = 8.74, *P*=0.54 Model 2: HL(T): χ^2^ = 9.14, *P*=0.55 Model 3: HL(T): χ^2^ = 9.01, *P*=0.44 Model 4: HL(T): χ^2^ = 9.65, *P*=0.37
Liu (2024) (25)	**-**	**-**	Models for 5^th^ GA: AUC=0.671 (0.601-0.740) Models for 6^th^ GA: AUC=0.796 (0.734-0.857) Models for 7^th^ GA: AUC=0.872 (0.814-0.930) Models for 8^th^ GA: AUC=0.871 (0.789-0.953) Models for 9^th^ GA: AUC=0.813 (0.679-0.947)	–	–
Mu (2024) (21)	–	Geographical validation	AUC(T): 0.767 (0.725-0.808), AUC(V): 0.738 (0.665-0.810)	✓	HL(T): *P*=0.491, HL(V): *P*=0.076
Mu (2024) (23)	10-fold cross-validation with bootstrap	Temporal validation	AUC(T): 0.707 (0.651-0.7639), AUC(V): 0.809	✓	HL(T): χ^2^ = 6.428, *P*=0.599
Ou (2024) (17)	Random split data (7:3)	–	AUC(T): 0.853 (0.796-0.911), AUC(V): 0.875 (0.763-0.986)	✓	Calibration intercept=0.000, calibration slop=1.00 HL: *P*=6.068 Nagelkerke pseudo-R^2^ = 0.442
Wu (2023) (30)	–	–	AUC: 0.814 (0.709-0.919)	–	–
Yang (2024) (24)	Random split data (3:1)	–	AUC(T): 0.777 (0.690-0.853), AUC(V): 0.781 (0.702-0.843)	✓	HL(T): *P*=0.559, HL(V): χ^2^ = 7.427, *P*=0.505
Youssef (2022)* (19)	–	–	Brigham model: C-statistic=0.55 (0.512-0.59) Updated Brigham model: C-statistic=0.57 (0.53-0.62)	✓	Brigham model: calibration intercept=-0.46 (-0.62, -0.31), calibration slop=0.42 (0.11-0.73)
Zhang J (2024) (18)	–	–	AUC: 0.81 (0.697-0.923)	✓	HL: *P*=0.238
Zhang Z (2024) (26)	–	–	AUC 0.809 (0.735-0.880)	–	–

*Youssef (2022): This is an external validation of previously established Brigham model, using completely independent data.

### 3.7 Model validation

Model validation methods varied across studies (**Table 4**). Internal validation was the most common, including random splitting data (n=3) (17, 20, 24), bootstrapping (n=2) (28, 29), and cross-validation (n=1) (23). Among these, 3 studies demonstrated high consistency in AUC between training and validation datasets (difference ≤ 0.02) (17, 24, 28), while one study showed significant discrepancy (difference 0.09~0.11) (20). External validation was reported in only 3 studies (13%) (19, 21, 23), with 2 demonstrating similar or improved performance in the validation set (21, 23). However, one study found poor performance even after updating the model (19). Notably, 7 studies did not perform validation, which could affect model reliability and generalizability.

### 3.8 ROB assessment

All models assessed by PROBAST showed high ROB (**Supplementary Table 3**), indicating significant methodological shortcomings in development/validation, raising concerns about their real-world performance. In the predictor domain, 7 studies had high ROB (16, 19–23, 28), with one study having unclear risk (26) due to missing details on predictors. In the outcome domain, 11 studies showed high ROB (16, 18, 19, 21–23, 25–29) due to lack of blinding in outcomes/predictors evaluations. In the analysis domain, 11 studies had high ROB, with 9 failing to meet the EPV criterion due to small sample sizes (17, 18, 21–24, 26, 29, 30). Also, 7 studies showed issues with overfitting, underfitting, and performance optimism, and lacked internal/external validation (16, 18, 19, 22, 26, 27, 30). For applicability, 12 studies indicated low ROB. However, 3 studies had unclear applicability, with 2 in the predictor domain (16, 26), and one in the outcome domain due to unclear definition of ‘pregnancy loss’ (29).

## 4 Discussion

This systematic review identified 15 studies describing 22 RPL risk prediction models. Despite the models showed moderate to excellent predictive performance, they were all assessed to have high ROB due to methodological deficiencies.

Utilizing the PROBAST tool, this study found that the model exhibited low applicability concerns (e.g., good alignment with the target population, predictors, and outcomes) but carried a high overall ROB, primarily reflected in domains such as predictor selection, outcome definition, and analysis methods. This contradictory result suggests that, although the models are theoretically suitable for the current clinical setting in its design, methodological flaws during their development may lead to deviations of predicted values from true risks, potentially affecting the models’ decision-support value in practice. It is also important to note that the PROBAST assessments relies on reporting completeness. Despite our efforts to reconstruct model development details through data verification, there may still be unidentified sources of bias (e.g., unrecorded measurement errors in variables).

Additionally, inconsistencies in defining the RPL population further challenge model generalizability. Most studies included in this review defined RPL as two or more pregnancy losses, but there were differences regarding gestational age and the requirement for consecutive pregnancy losses. The primary goal of prediction models in the medical field is to support informed decision-making. Therefore, it is important to clearly define the target population in order to evaluate the performance of the developed model and ensure users understand its applicability. Moreover, 10 studies were deemed to have high ROB due to model overfitting. Overfitting is a common challenge in constructing multivariable prediction models. The coexistence of high AUCs and high ROB may stem from overfitting in small samples, where models capture noise rather than true biological signals, inflating performance in training sets but failing in external validation (32). To mitigate these risks, widely accepted guidelines recommend a minimum of 10 EPV to reduce overfitting and improve generalization, thereby minimizing bias (33, 34). However, 8 studies reported EPV<10 (18, 21–24, 26, 29, 30), and 2 studies were unable to calculate EPV (17, 20). Importantly, low EPV can lead to inaccurate effect estimates and statistical instability, which threatens model validity and may result in poor performance on new datasets (35, 36). Therefore, restricting their application to narrowly defined subpopulations could mitigate misclassification risks until methodological refinements are achieved. Future research should adhere established guidelines for constructing models to enhance the model’s predictive power and generalizability.

In this review, the most frequently included predictive variables were maternal age, number of previous pregnancy losses, and maternal BMI, which have been consistently incorporated into RPL prediction models over the past five years. Their association with RPL abortion has been extensively validated. Advanced maternal age is associated with increased risk of chromosomal abnormalities and reduced endometrial receptivity (37). Elevated maternal BMI has been linked to insulin resistance, chronic inflammation, and impaired implantation (38). A greater number of pregnancy losses often reflects persistent underlying risk factors, such as anatomical, immunological, or coagulation-related issues, which could affect the success rate of subsequent pregnancies and embryo implantation (39). In addition to these strong patient-related factors, a growing number of studies have explored immunological and metabolic/endocrine predictors. For instance, ANA and ACA suggest underlying autoimmune activation that could interfere with embryo implantation or promote placental thrombosis (40, 41). Increased concentrations of C4 could indicate an overly active immune reaction, which may result in harm to the fetal-placental unit (42). On the endocrine side, abnormal levels of progesterone or TSH could indicate luteal phase deficiency or subclinical hypothyroidism, both of which are associated with adverse pregnancy outcomes (43). These findings highlight the multifactorial nature of RPL and the importance of integrating diverse predictor domains in model development.

Furthermore, one model included paternal factors (age and BMI) and the mode of conception as predictors for assessing RPL pregnancy outcomes (28). These couple-related predictors are rarely explored but may be important contributors to reproductive outcomes. For example, while natural conception is commonly influenced by basic health factors like age and BMI, patients undergoing assisted reproductive technology (ART) face additional challenges such as ovarian dysfunction and embryo quality (44, 45). However, over 70% of included studies did not specify whether pregnancies were naturally conceived or ART-mediated. Only two studies reported inclusion of ART conceptions, but these were analyzed together with natural conceptions without reporting pregnancy outcomes separately (17, 22). Given the rising prevalence of ART, future research should explore how conception methods interact with risk factors and should clearly report and stratify by conception method. This would help refine RPL risk models, improve their clinical applicability, and support more precise prognostic assessments.

Assessing the performance of a risk prediction model is crucial after its development. In this review, only 6 studies performed internal validation, and just 2 performed external validation. Although many studies demonstrated good discrimination in their training datasets, the lack of proper validation contributed to a high risk of bias, particularly in the analysis domain. This limitation undermines the reliability and clinical applicability of these models and highlights the need for improved data quality, validation strategies, and transparent reporting.

To our knowledge, this is the first systematic review to evaluate the risk prediction models for RPL pregnancy outcomes. Our review highlighted the methodological deficiencies in these studies, which could inform future research in constructing more robust, reliable models applicable across various clinical settings. Unlike previous reviews that primarily focus on English-language sources, our study also includes Chinese-language literature, offering a broader evaluation of existing RPL risk prediction models. However, due to heterogeneity across studies, such as differences in predictor variables, pregnancy outcome use, data sources, and analytical methods, only a narrative systematic review was conducted. Additionally, most of the included studies (12/15) were conducted in China, with only 2 from the Netherlands and 1 from Israel. This concentration in a single region raises the possibility of regional bias. Differences in population characteristics, healthcare practices, and resource availability across countries may influence both model performance and applicability. As a result, these models may not be directly applicable to broader or more heterogeneous populations. Future research should consider validating or adapting existing models in more diverse clinical settings to improve their generalizability and clinical utility.

Collectively, to translate these findings into clinical utility, future research must prioritize immediate and longer-term goals. Immediate priorities should focus on both internal and external validation of existing models across diverse populations, standardization of outcome definitions (e.g., gestational age), and adherence to EPV criteria and TRIPOD (Transparent Reporting of a multivariable prediction model for Individual Prognosis Or Diagnosis) statement (46) during model development. These steps are critical to confirm generalizability, reduce measurement bias, and prevent overfitting—issues that directly undermine current models’ reliability. Simultaneously, longer-term strategies could explore adaptive model architectures capable of dynamically integrating emerging data, thereby maintaining relevance as clinical practices evolve. This approach would enhance accuracy while reducing reliance on manual recalibration. By addressing these challenges, the developed risk prediction models can better support clinical practice in managing patients with RPL, ultimately leading to more personalized, timely, and effective interventions that improve patient outcomes.

## 5 Conclusions

The findings suggest that current risk prediction models for RPL pregnancy outcomes have a high ROB in clinical applications. Future research should prioritize rigorous model construction and validation processes to provide more reliable and effective tools for clinical practice.

## Data Availability

The original contributions presented in the study are included in the article/**Supplementary Material**. Further inquiries can be directed to the corresponding author.
