# Supplementary material for: Risk prediction models for pregnancy outcomes in recurrent pregnancy loss: a narrative systematic review
Source: Front Endocrinol (Lausanne). 2025 May 29;16:1582156. doi: 10.3389/fendo.2025.1582156 (PMC12158742; doi:10.3389/fendo.2025.1582156)
Supplement: Supplementary file 1 [file DataSheet1.docx]

**Supplementary data**

**Supplementary Figure 1.** Geographic distribution of the included studies.


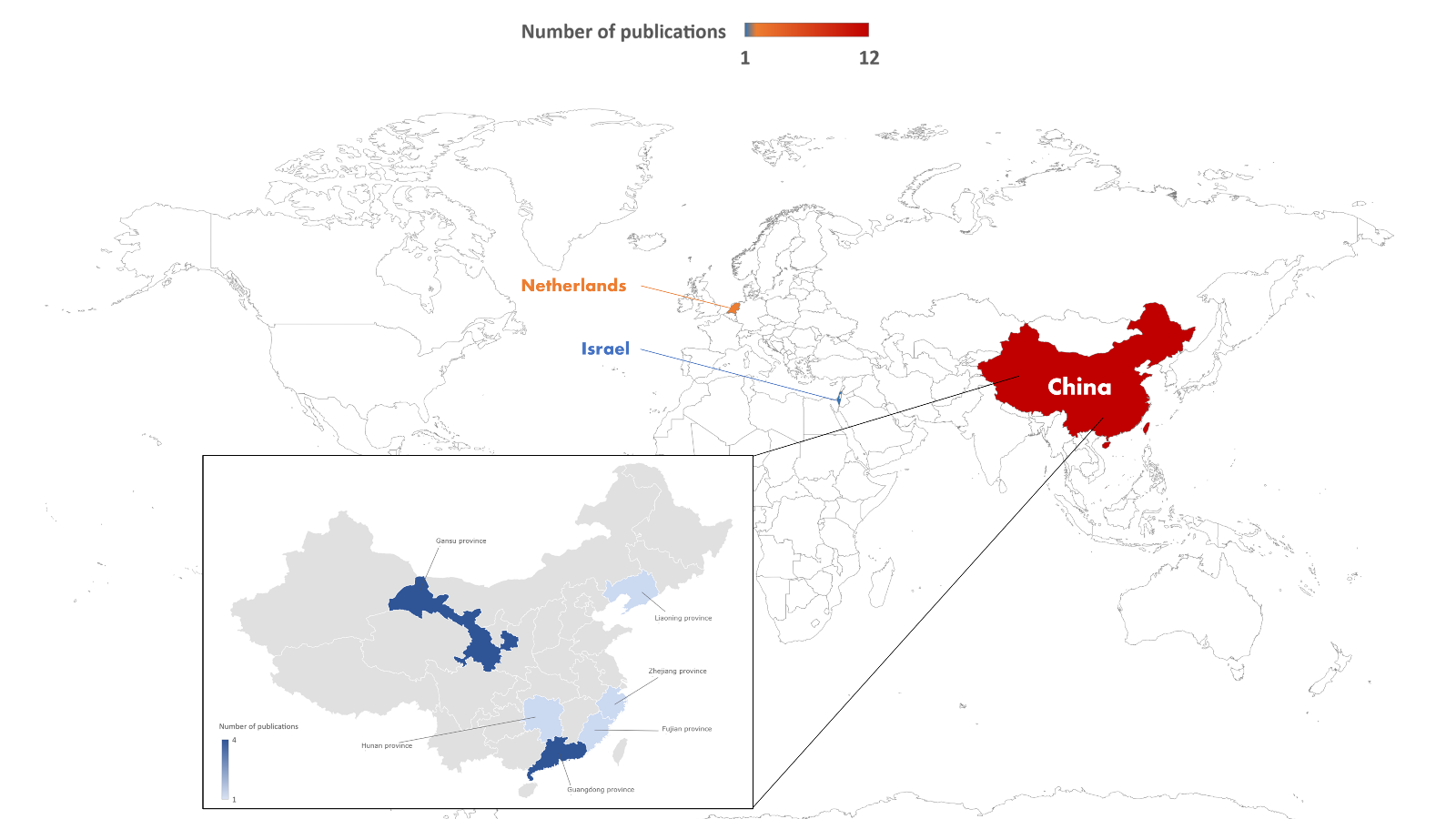


**Supplementary Figure 2.** Distribution of predictor variables in the model.

**
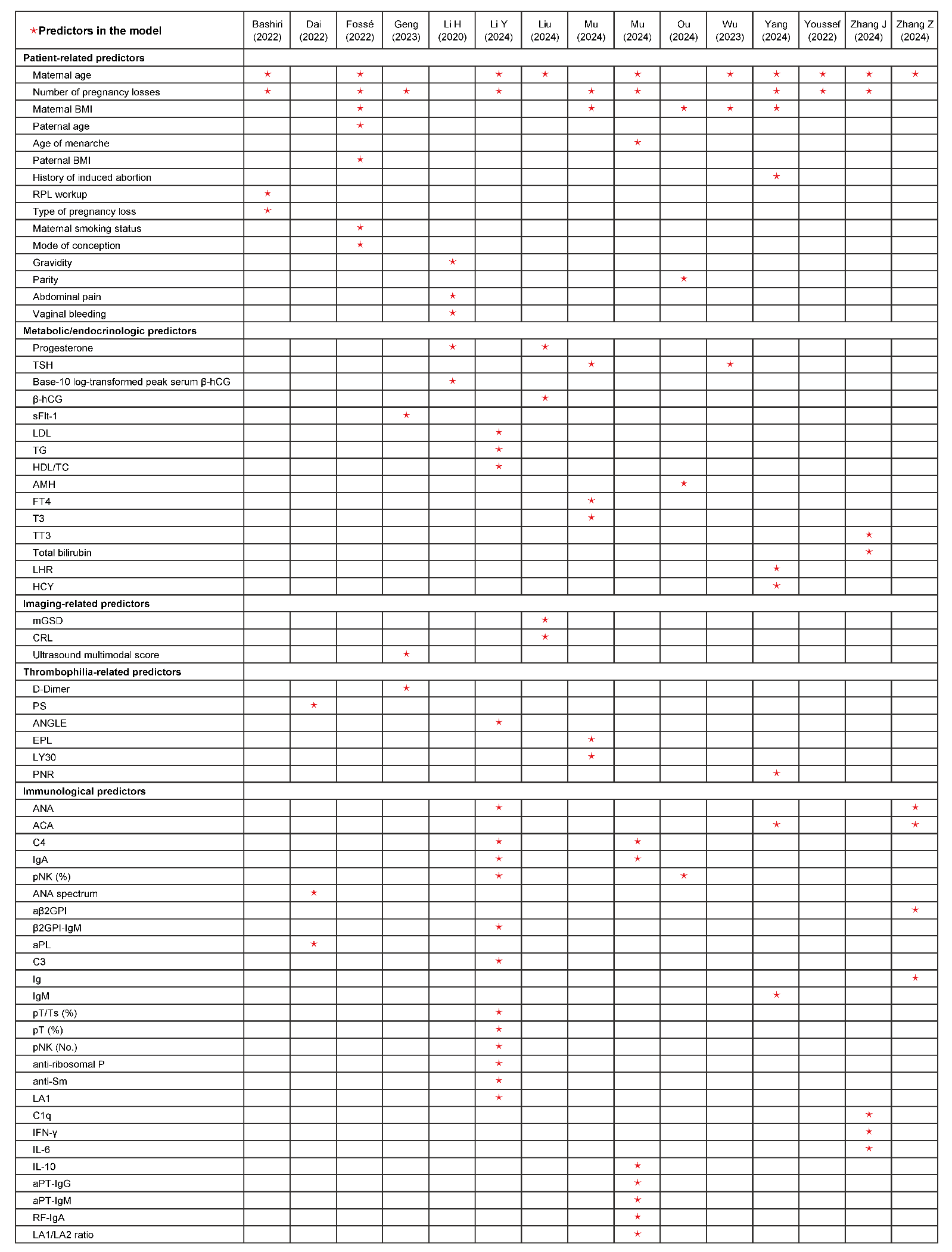
**

*🟉: Predictors in the final model. ANA, antinuclear antibody; PS, protein S; aPL, anti-phospholipid antibody; BMI, body mass index; sFlt-1, soluble vascular endothelial growth factor receptor-1; β-hCG, β human chorionic gonadotropin; C3, complement 3; C4, complement 4; LDL, low-density lipoprotein; HDL, high-density lipoprotein; TC, cholesterol; TG, triglyceride; pT, peripheral T cells; Ts, suppressor T cells; pNK, peripheral natural killer cells; pT (%), percentages of peripheral T cells; pNK (%), percentage of pNK; pNK (No.), number of pNK; β2GPI, β2 glycoprotein I antibodies; aβ2GPI, anti-β2 glycoprotein I antibody; Ig, immunoglobulin; IgA, immunoglobulin A; IgG, immunoglobulin G; IgM, immunoglobulin M; mGSD, mean of the gestational sac diameter; CRL, crown-rump length; BMI, body mass index; T3, triiodothyronine; FT4, free thyroxine; TSH, thyroid stimulating hormone; LY30, an actual percentage of clot lysis at 30 minutes after the maximum amplitude on TEG tracing; EPL, an estimated percentage of clot lysis at 30 minutes after the maximum amplitude on TEG tracing; ANGLE, angle Alpha; IL-10, interleukin-10; aPT, anti-prothrombin antibody; RF, rheumatoid factor; LA, lupus anticoagulant; LA1, screening test; LA2, LA confirmatory test; AMH, anti-Müllerian hormone; ACA, anti-cardiolipin antibody; HCY, homocysteine; PNR, platelet to neutrophilic ratio; LHR, low-density lipoprotein to high-density lipoprotein ratio; IL-6, interleukin-6; TT3, total triiodothyronine; IFN-γ, interferon-γ; C1q, complement component 1q.*

**Supplementary Figure 3.** Frequency of inclusion of predictive variables across categories by publication year.

**
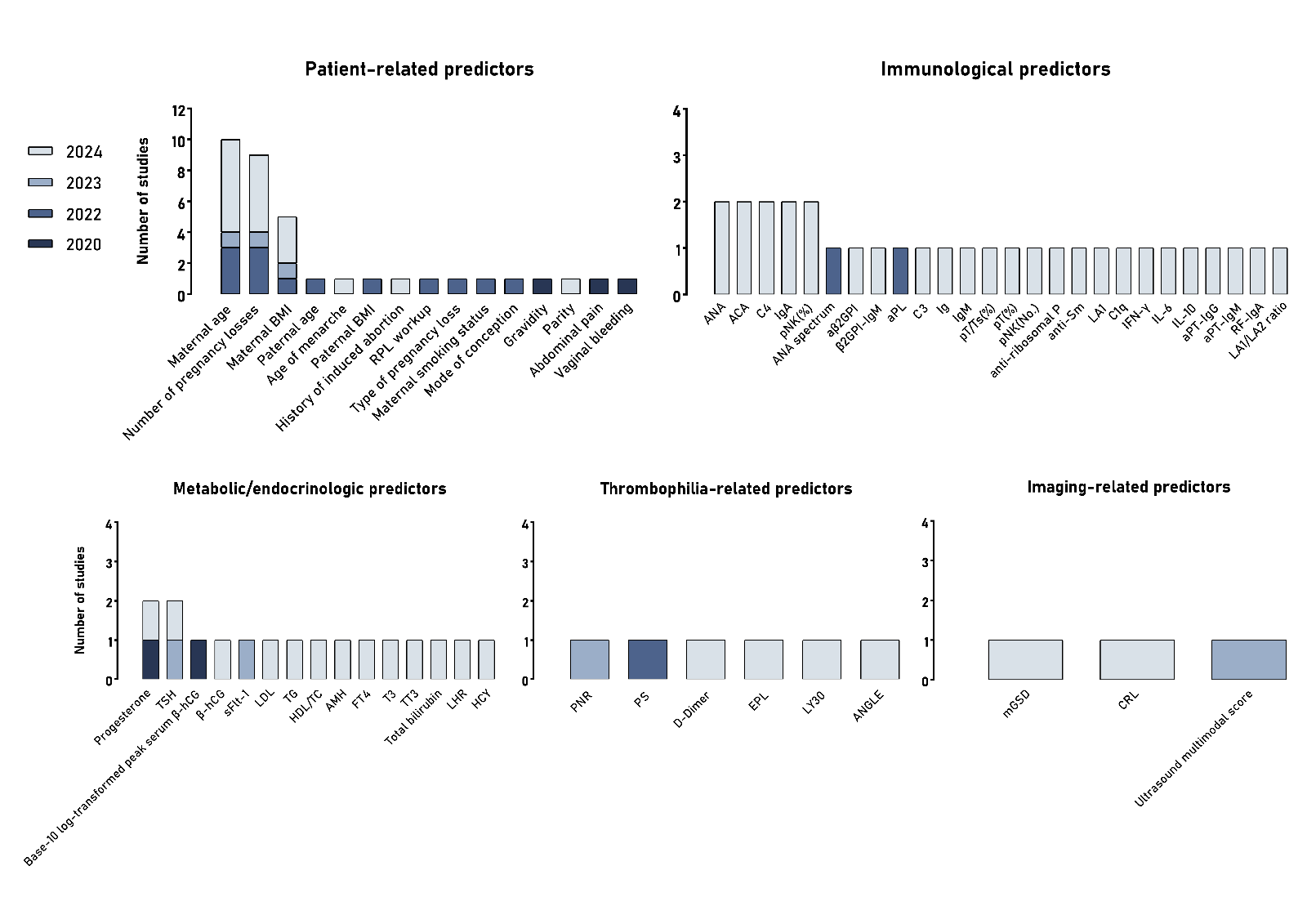
**

*ANA, antinuclear antibody; PS, protein S; aPL, anti-phospholipid antibody; BMI, body mass index; sFlt-1, soluble vascular endothelial growth factor receptor-1; β-hCG, β human chorionic gonadotropin; C3, complement 3; C4, complement 4; LDL, low-density lipoprotein; HDL, high-density lipoprotein; TC, cholesterol; TG, triglyceride; pT, peripheral T cells; Ts, suppressor T cells; pNK, peripheral natural killer cells; pT (%), percentages of peripheral T cells; pNK (%), percentage of pNK; pNK (No.), number of pNK; β2GPI, β2 glycoprotein I antibodies; aβ2GPI, anti-β2 glycoprotein I antibody; Ig, immunoglobulin; IgA, immunoglobulin A; IgG, immunoglobulin G; IgM, immunoglobulin M; mGSD, mean of the gestational sac diameter; CRL, crown-rump length; BMI, body mass index; T3, triiodothyronine; FT4, free thyroxine; TSH, thyroid stimulating hormone; LY30, an actual percentage of clot lysis at 30 minutes after the maximum amplitude on TEG tracing; EPL, an estimated percentage of clot lysis at 30 minutes after the maximum amplitude on TEG tracing; ANGLE, angle Alpha; IL-10, interleukin-10; aPT, anti-prothrombin antibody; RF, rheumatoid factor; LA, lupus anticoagulant; LA1, screening test; LA2, LA confirmatory test; AMH, anti-Müllerian hormone; ACA, anti-cardiolipin antibody; HCY, homocysteine; PNR, platelet to neutrophilic ratio; LHR, low-density lipoprotein to high-density lipoprotein ratio; IL-6, interleukin-6; TT3, total triiodothyronine; IFN-γ, interferon-γ; C1q, complement component 1q.*

**Supplementary Table 1** Full search strategies for all included databases (as of August 2, 2024)

| **PubMed** | |
| --- | --- |
| #1 | "abortion, habitual"[MeSH Terms] OR "habitual abortion*"[Title/Abstract] OR "recurrent abortion*"[Title/Abstract] OR "recurrent miscarriage*"[Title/Abstract] OR "recurrent early pregnancy loss"[Title/Abstract] OR "recurrent pregnancy loss*"[Title/Abstract] OR "recurrent spontaneous abortion*"[Title/Abstract] |
| #2 | ("nomograms"[MeSH Terms] OR "machine learning"[MeSH Terms] OR "deep learning"[MeSH Terms] OR "support vector machine"[MeSH Terms] OR "logistic models"[MeSH Terms] OR "random forest"[MeSH Terms] OR "risk assessment"[MeSH Terms] OR "nomogram"[Title/Abstract] OR "predict* model"[Title/Abstract] OR "machine learning"[Title/Abstract] OR "support vector machine"[Title/Abstract]) |
| #3 | ((review[Publication Type]) OR (systematic review[Publication Type])) OR (meta analysis[Publication Type]) |
| #4 | ((#1 AND #2) NOT #3) AND ((excludepreprints[Filter]) AND (humans[Filter]) AND (chinese[Filter] OR english[Filter])) |
| **Embase** | |
| #1 | ('recurrent abortion'/exp OR 'recurrent abortion' OR 'recurrent abortion':ti,ab,kw) AND ([article]/lim OR [article in press]/lim) AND ([chinese]/lim OR [english]/lim) AND [humans]/lim |
| #2 | ('nomogram'/exp OR 'nomogram' OR 'nomogram':ab,kw,ti OR 'predictive model'/exp OR 'predictive model' OR 'predictive model':ab,kw,ti OR 'machine learning'/exp OR 'machine learning' OR 'machine learning':ab,kw,ti OR 'support vector machine'/exp OR 'support vector machine' OR 'support vector machine':ab,kw,ti OR 'risk model'/exp OR 'risk model' OR 'risk model':ab,kw,ti OR 'deep learning'/exp OR 'deep learning' OR 'deep learning':ab,kw,ti OR 'logistic regression analysis'/exp OR 'logistic regression analysis' OR 'logistic regression analysis':ab,kw,ti OR 'gradient boosting machine'/exp OR 'gradient boosting machine' OR 'gradient boosting machine':ab,kw,ti OR 'random forest'/exp OR 'random forest' OR 'random forest':ab,kw,ti OR 'sparse coding':ab,kw,ti) AND ([article]/lim OR [article in press]/lim) AND ([chinese]/lim OR [english]/lim) AND [humans]/lim |
| #3 | #1 AND #2 |
| **Web of Science** | |
| #1 | "(((((((TS=(recurrent abortion*)) OR TS=(recurrent miscarriage)) OR TS=(recurrent pregnancy loss*)) OR TS=(recurrent spontaneous abortion*)) OR TS=(repeated abortion*)) OR TS=(repeated miscarriage*)) OR TS=(repeated spontaneous abortion*)) OR TS=(repeated pregnancy loss*) and Preprint Citation Index (Exclude – Database)" |
| #2 | "((((((((((((TS=(nomogram*)) OR TS=(machine learning)) OR TS=(predict* model)) OR TS=(predict* tool)) OR TS=(deep learning)) OR TS=(random forest)) OR TS=(support vector machine)) OR TS=(SVM)) OR TS=(gradient boosting machine)) OR TS=(GBM)) OR TS=(XGboost)) OR TS=(logistic regression)) OR TS=(sparse coding) and Preprint Citation Index (Exclude – Database)" |
| #3 | "#1 AND #2 and Preprint Citation Index (Exclude – Database) and Article (Document Types) and Web of Science Core Collection (Database) and English or Chinese (Languages) " |
| **CNKI** | |
| #1 | 主题：复发性流产(recurrent abortion) + 反复妊娠丢失(recurrent pregnancy loss) |
| #2 | 主题：预测模型(prediction model) + 列线图(nomogram) + 机器学习(machine learning) |
| #3 | #1 AND #2 |
| **CMAJ** | |
| #1 | TM=复发性流产(recurrent abortion) OR 反复妊娠丢失(recurrent pregnancy loss) |
| #2 | TM=预测模型(prediction model) OR 预测(prediction) OR 列线图(nomogram) OR 机器学习(machine learning) |
| #3 | #1 AND #2 |

*CNKI*=China National Knowledge Infrastructure, *CMAJ*=China Medical Association Journals

**Supplementary Table 2** Inclusion and exclusion criteria of the included studies

| First author (year) | Inclusion criteria | Exclusion criteria |
| --- | --- | --- |
| Bashiri (2022) | Pregnant women with RPL (i.e. two or more pregnancy losses) | - |
| Dai (2022) | Patients with two or more consecutive abortions before 28 weeks of gestation | Ectopic pregnancy or embryonic chromosome abnormality; couples with chromosomal abnormalities; uterine anatomic abnormalities or organic pathological changes; consanguineous marriage; familial genetic diseases; coagulation, liver or kidney dysfunction; RHD or CHD. |
| Fossé (2022) | Couples with at least two pregnancy losses before 24 weeks of gestation (following the definition of the ESHRE guideline on RPL) in the current relationship | Couples with pregnancy losses after oocyte or sperm donation; couples with an identified underlying condition for RPL, including uterine anomalies, thyroid abnormalities (TPO and TSH levels), acquired thrombophilia (aPLs), and parental chromosomal translocations. |
| Geng (2023) | Early pregnant patients with a history of URPL who met the following criteria: (1) a history of ≥3 spontaneous abortions, regular menstrual cycle, normal menstrual flow, the cause of miscarriage excludes genetic, infectious, endocrine, immune, and couple’s chromosomal karyotype abnormalities, and ruled out organic abnormalities of reproductive organs; (2) a singleton pregnancy with gestation week <12 weeks; (3) receiving ultrasound multimodal monitoring with complete data; (4) signed informed consent. | Combined with AIDS, hepatitis or other infectious diseases; history of drug use and alcohol abuse during pregnancy; combined with uterine polyps, fibroids, or cervical insufficiency; history of thrombosis or use of anticoagulant or pro-fibrinolytic drugs within the past two months; conceived through ART. |
| Li H (2020) | A history of RPL with a singleton pregnancy and positive EHM detected by ultrasound before 14 full weeks’ gestation | Irregular menstrual cycles, twin or multiple pregnancies, ectopic pregnancy, trophoblastic disease, induced abortion because of fetal dysplasia or reproductive history, and uncertainty of the early pregnancy outcome. |
| Li Y (2024) | Women aged between 18 and 44 years who had experienced URPL, visited hospital with a complaint of URPL and experienced another pregnancy after the hospital visit | Anatomic abnormalities of the uterus, chromosomal abnormalities (of either parent), endocrine disorders, reproductive tract infections, autoimmune diseases such as SLE, UCTD and RA, and those with missing data. |
| Liu (2024) | Women with RPL aged between 18 and 45 years old were included. All these patients underwent a comprehensive assessment prior to their initial visit for preconception planning or at their first visit in our clinic during the early stages of pregnancy, including transvaginal ultrasound to exclude uterine structural abnormalities, thyroid function test, antiphospholipid syndrome related antibodies, and other RPL related tests according to European Guideline for RPL. | Patients who were lost to follow up, had no plan to conceive any more, and those who were pregnant in the first trimester at the last follow-up; patients with multiple pregnancies and those who conceived a second time. |
| Mu (2024) | Patients aged ≥18 years and experienced a history of at least two pregnancy losses before 24th weeks of gestation | Parental chromosomal abnormalities; autoimmune diseases such as APS, SLE and SS; hyperthyroidism, hypothyroidism, and subclinical hypothyroidism; PCOS; uterine anatomic abnormalities; autoantibody test positive such as aPLs, aβ2GPI, LA and ANAs; infertility; adverse pregnancy outcomes such as molar pregnancy and ectopic pregnancy; lack of necessary follow-up information; absence of necessary pre-pregnancy thyroid function or TEG tests; no fertility plan. |
| Mu (2024) | Patients with a history of two pregnancy losses occurring before the 24th gestational week and aged ≥18 years | Abnormal chromosome karyotype in a couple; APS or other autoimmune diseases (such as thyroid autoimmune disease, SLE, SS, connective tissue disease); abnormal thyroid function; PCOS; congenital uterine malformations; no fertility plan; diagnosis of infertility; adverse obstetric outcomes such as hydatid mole, ectopic pregnancy and congenital disability; no complete records of pre-pregnancy immunological parameters tests; with an unknown pregnancy outcome. |
| Ou (2024) | URPL was diagnosed among women with RPL without known causes based on the ESHRE guidelines | A previous pregnancy loss after 12 gestational weeks (because the causative factors for second vs. first trimester pregnancy loss are different); lack of consent; positive for ANAs or antithyroid antibody; no conception within a year after initial blood tests or until study end, whichever was sooner; unwilling to have a blood sample taken for pNK cell level measurement during pregnancy; and the use of immunologic medication before or after three months of pregnancy. |
| Wu (2023) | A history of two or more consecutive spontaneous abortions (including biochemical pregnancies); aged 20~48 years. | Chromosomal abnormalities or severe genetic diseases in either partner; severe semen abnormalities in the partner, such as oligospermia, asthenospermia, or teratospermia; acute infectious diseases or severe primary diseases of the heart, brain, lungs, liver, kidneys, blood system, or psychiatric disorders; inability to complete follow-up due to cognitive impairment or poor compliance; incomplete clinical data. |
| Yang (2024) | Patients have experienced at least two history of pregnancy loss that meets the diagnostic criteria of the ESHRE; aged 18~42 years | Patients who were lost to follow-up and who were not yet pregnant; subsequent pregnancy outcomes are ectopic pregnancy, hydatidiform mole, dysplasia, and current pregnancy <10 weeks; subsequent pregnancies were ART and twin pregnancies. |
| Youssef (2022) | Patients without the presence of an identifiable cause for the pregnancy losses, according to the ESHRE guideline | - |
| Zhang J (2024) | Patients with two or more consecutive pregnancy losses before 24 weeks of gestation | Ectopic pregnancy and molar pregnancy and incomplete clinical records; ultrasound-confirmed uterine malformation; abnormal karyotype; hormone or metabolic disorder other than thyroid dysfunction; acquired thrombotic tendency; known clinical autoimmune diseases; chronic diseases; severe reproductive system infection. |
| Zhang Z (2024) | All patients were treated with CsA, with 25 mg bid per day from the preparation for pregnancy until 12 weeks of gestation or abortion. The preparation of pregnancy will be followed by a one-year pre-pregnancy phase, and if participants do not become pregnant within that year, their treatment and participation will be terminated. The additional inclusion criteria are as follows: (1) Continued spontaneous abortion or unexplained fetal arrest and death occurred in utero more than two times; and (2) neither partner was consanguineous nor had normal karyotypes. | Incomplete clinical data |

RPL=recurrent pregnancy loss, URPL=unexplained recurrent pregnancy losss, ESHRE=European Society of Human Reproduction and Embryology, AIDS= acquired immune deficiency syndrome, ART=assisted reproduction technology, EHM=embryonic heart motion, RHD=rheumatic heart disease, CHD=congenital heart disease, TPO=antithyroid peroxidase, TSH=thyroid-stimulating hormone, aPLs=anti-phospholipid antibodies, SLE=systematic lupus erythematosus, UCTD=undifferentiated connective-tissue disease, RA=rheumatoid arthritis, APS=antiphospholipid syndrome, SS=Sjögren’s syndrome, PCOS=polycystic ovary syndrome, aβ2GPI=anti-β2 glycoprotein antibody, LA=lupus anticoagulant, ANAs=antinuclear antibodies, TEG=thromboelastograph, pNK=peripheral natural killer cells.

**Supplementary Table 3** Tabular presentation for PROBAST results of included studies

| Study ID | Type of model | Risk of bias | | | | Applicability | | | Overall | |
| --- | --- | --- | --- | --- | --- | --- | --- | --- | --- | --- |
|  |  | **Participants** | **Predictors** | **Outcome** | **Analysis** | **Participants** | **Predictors** | **Outcome** | **ROB** | **Applicability** |
| Bashiri (2022) | A | ? | + | - | - | + | + | + | - | + |
| Dai (2022) | A | + | - | - | - | + | ? | + | - | ? |
| Fossé (2022) | B | + | - | - | + | + | + | + | - | + |
| Geng (2023) | B | + | + | - | - | + | + | - | - | - |
| Li H (2020) | A | + | - | - | - | + | + | + | - | + |
| Li Y (2024) | B | + | - | + | + | + | + | + | - | + |
| Liu (2024) | A | + | + | - | + | + | + | + | - | + |
| Mu (2024) | B | + | - | - | - | + | + | + | - | + |
| Mu (2024) | B | + | - | - | - | + | + | + | - | + |
| Ou (2024) | B | + | + | + | - | + | + | + | - | + |
| Wu (2023) | A | + | + | + | - | + | + | + | - | + |
| Yang (2024) | B | + | + | + | - | + | + | + | - | + |
| Youssef (2022) | C | + | - | - | + | + | + | + | - | + |
| Zhang J (2024) | A | + | + | - | - | + | + | + | - | + |
| Zhang Z (2024) | A | + | ? | - | - | + | ? | + | - | ? |

A indicates “development only”; B indicates “development and validation in the same publication”, C indicates “validation only”.

+ indicates low ROB/low concern regarding applicability.

- indicates high ROB/high concern regarding applicability.

? indicates unclear ROB/unclear concern regarding applicability.
